# Conduction Block and Nerve Cross-Sectional Area in Multifocal Motor Neuropathy

**DOI:** 10.3389/fneur.2019.01055

**Published:** 2019-10-09

**Authors:** Yi Li, Jingwen Niu, Tanxin Liu, Qingyun Ding, Shuang Wu, Yuzhou Guan, Liying Cui, Mingsheng Liu

**Affiliations:** ^1^Department of Neurology, Peking Union Medical College Hospital, Chinese Academy of Medical Sciences, Beijing, China; ^2^Department of Medical English, Peking University Health Science Center, Beijing, China

**Keywords:** multifocal motor neuropathy, conduction block, cross-sectional area, ultrasound, electromyography

## Abstract

**Introduction:** Motor nerve conduction block (CB) is the main electrophysiological feature of multifocal motor neuropathy (MMN). Increased cross-sectional area (CSA) can be detected by nerve ultrasound in MMN. In this study, we aim to analyze the correlation between CB and CSA in MMN.

**Methods:** Twelve patients with MMN were recruited. Ultrasonography tests and motor nerve conduction studies (NCSs) were performed on median and ulnar nerves simultaneously. CSA was measured at 10 consecutive sites on those nerves, meanwhile nerves were traced continuously and recorded thoroughly under ultrasound.

**Results:** In motor NCSs, 12 definite CB and 12 probable CB areas were detected across standard segments of median and ulnar nerves. With ultrasound studies, increased CSA was detected at 36 sites. There were 9 standard segments with CB and increased CSA, 15 segments with CB but normal CSA, and 27 segments with increased CSA but no CB.

**Discussion:** In MMN, motor nerve CB was not always consistent with increased CSA.

## Introduction

Multifocal motor neuropathy (MMN) was first described in 1988 (1) as a purely motor neuropathy affecting multiple motor nerves with conduction block (CB). Motor CB is the core electrophysiological hallmark for the diagnosis of MMN. Nerve ultrasound can supply the morphological features of peripheral nerves. The multifocal enlargement of peripheral nerves or cervical roots in nerve cross-sectional areas (CSAs) has been reported in MMN (2–4). Kerasnoudis et al. (5) reported a correlation between compound motor action potentials (CMAPs) and CSAs of the median nerve in the upper arm (*r* = 0.851, *p* < 0.001). Beekman et al. (6) found that sonography studies showed increased nerve CSA compatible with conduction abnormalities more than expected on purely clinical grounds. Moreover, some sites exhibited nerve enlargement without CB. Multifocal CBs were distributed along the nerve in MMN; however, those studies only measured a few predetermined sites, providing limited morphologic information. In this study, the use of consecutive scanning along the nerve and measuring CSA at multiple sites based on ultrasound allowed a more accurate correlation between conduction block and increased CSA in MMN.

## Methods

### Subjects

Between December 2014 and May 2018, 12 MMN patients were consecutively recruited from Peking Union Medical College Hospital according to criteria proposed by the AANEM (7). A same number of healthy controls (HC), matched by age (±1 years), were enrolled as controls. All patients and healthy controls underwent a standardized clinical examination including muscle strength testing of the wrist, thumb and finger flexion, opponens pollicis, abductor pollicis brevis, finger spreading, and adductor pollicis, together with sensory testing. Clinical examinations, electromyogram, and nerve ultrasound studies were performed on the same day. The ethics committee of Peking Union Medical College Hospital approved our study protocol, and all patients signed an informed consent form in accordance with the Declaration of Helsinki.

### Nerve Conduction Studies

Motor nerve conduction studies (NCSs) were performed on all subjects on the bilateral median and ulnar nerves with percutaneous supramaximal nerve stimulation while recording CMAPs with 10-mm disk electrodes. Standard segments were defined as wrist to elbow and elbow to axilla for the median nerve, and as wrist to below elbow and upper elbow to axilla for the ulnar nerve. An inching technique (stimulating along the course of the nerve in 2-cm increments) was performed across some standard segments with a partial conduction block, detecting the exact site of CB, along with a consecutive ultrasound test across the same segment. The CB diagnosis of standard segments and the inching technique were performed according to criteria suggested by the AANEM (7). To include only true conduction block, distal CMAP had to be 1 mV. Room temperature was maintained to ensure that the skin temperature remained at >31°C. Technicians were blinded to patient information.

### Ultrasound

Ultrasonography tests were performed via nerve tracing from wrist to axilla on the bilateral median and ulnar nerves with a 10 MHz linear array transducer (GE LOGIQ e, USA). In order to eliminate artificial increase of nerve size, the use of zoom magnification was not allowed for these measurements. The initial settings were kept constant during all examinations including depths. The transducer was kept perpendicular to the nerve at an angle selected to obtain the smallest and brightest image. The CSAs at the predetermined sites on each nerve were measured by tracing just inside the hyperechoic rim of the nerve. Ten predetermined sites were measured on each nerve according to a previous report from our laboratory (8). For the median nerve, the 10 sites included the outlet of the carpal tunnel (M1), the middle point of the wrist crease (M2), the inlet of the carpal tunnel (M3), 4 cm proximal to the wrist crease (M4), the middle between the wrist crease and elbow (M5), the entrance into the pronator teres (M6), the elbow (M7), 4 cm above the elbow (M8), 8 cm above the elbow (M9), and the axilla (M10). For the ulnar nerve, the 10 sites included the wrist (U1), 4 cm proximal to the wrist (U2), the departing point from the ulnar artery (U3), alongside the muscle belly of the flexor carpi ulnaris (U4), the outlet of the cubital tunnel (U5), inside the cubital tunnel (U6), the inlet of the cubital tunnel (U7), 4 cm proximal to the inlet of the cubital tunnel (U8), 8 cm proximal to the inlet of the cubital tunnel (U9), and the axilla (U10). Except for the abovementioned sites, measurements were also taken at any other sites of enlargement. CSA enlargement was referenced to the normative values in our laboratory (in the median nerve, forearm-elbow segments were ≤ 10 mm^2^, and elbow-axilla segments were ≤ 9 mm^2^; in the ulnar nerve, both forearm and arm segments were ≤ 6 mm^2^). After CSA measurement, the nerve was again traced continuously and recorded thoroughly. Common compressive neuropathies resulting in nerve enlargement had been excluded from study. Ultrasonographers were blinded to patient information.

### Statistics

The CSAs of MMN showed a non-normal distribution. The Mann-Whitney *U*-test was used to compare MMN and healthy controls, and the difference in maximum CSAs between segments with CB and those without CB. Maximum CSA was defined as the maximal CSA across the standard segment. For all tests, a two-sided *P*-value of <0.05 was considered statistically significant.

## Results

### Clinical Features

Eight men and 4 women with a mean age of 43.7 years (range 21–62, SD 13.2), 12 healthy controls (mean age 43.6, range 28–57, SD 13.3, 8 men) were included in this study. The average disease duration was 65.3 (24–108) months. Mean height was 168 (155–186) cm and mean weight was 65.4 (56.5–88) kg. All patients were treatment-naive.

### Cross-Sectional Area (CSA)

**Figure 1:**
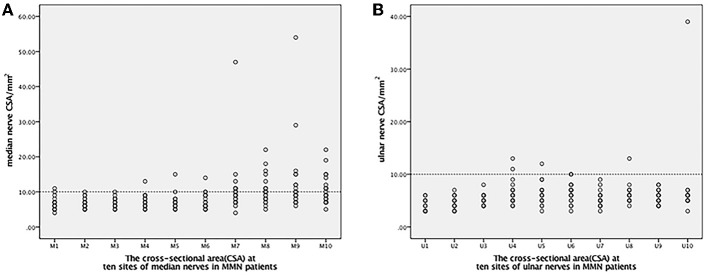
The distribution of 10 CSA sites in the median and ulnar nerves. The line of 10 mm^2^ was set up as distinguishing significant abnormalities CSA sites from the other segments. **(A)** For the median nerve: M1-the outlet of the carpel cannel, M2-the middle point of the wrist crease, M3-the inlet of the carpel cannel, M4-4 cm proximal to the wrist crease, M5-the middle point between the wrist crease and elbow, M6-the entrance into the pronator teres, M7-the elbow, M8-4 cm above the elbow, M9-8 cm above the elbow, M10-the axilla. **(B)** For the ulnar nerve: U1-the wrist, U2-4 cm above the wrist, U3-departing point from the ulnar artery, U4-alongside the muscle belly of the flexor carpi ulnaris, U5-the outlet of the cubital tunnel, U6-inside the cubital tunnel, U7-the inlet of the cubital tunnel, U8-4 cm proximal to the inlet of the cubital tunnel, U9-8 cm proximal to the inlet of the cubital tunnel, U10-axilla. CSA, cross-sectional area; MMN, multifocal motor neuropathy.

The CSA values for the median and ulnar nerves in MMN and HC at the 10 sites are shown in Table 1 and in Figure 1. The CSA enlargements were multifocal when compared with healthy controls. In median nerves, higher CSA values were mainly distributed in the forearm segment and upper arm segment. The below-elbow sites and upper-arm segment of ulnar nerves showed more obvious CSA enlargement. Interestingly, common sites of nerve compression, such as the carpal canal and cubital tunnel did not reveal a prevalent CSA increase in MMN patients comparing with healthy controls.

**Table 1:** CSA at different sites of median and ulnar nerves in MMN and HC.

	**M1**	**M2**	**M3**	**M4**	**M5**	**M6**	**M7**	**M8**	**M9**	**M10**
Median nerve CSA/mm^2^ Mean (P25–P75)	6.6 (5.0–7.0)	6.7 (6.0–7.0)	6.8 (6.0–8.0)	7.2 (6.0–8.0)	7.1 (6.0–8.0)	7.1 (6.0–9.0)	10.7 (7.0–11.0)	10.5 (8.0–11.0)	12.6 (8.0–12.0)	10.4 (7.0–12.0)
Healthy control CSA (mean ± SD)	6.1 (6.0–7.0)	6.5 (6.0–7.0)	6.4 (6.0–7.0)	5.9 (6.0–6.0)	5.7 (5.0–6.0)	5.6 (5.0–6.0)	8.7 (8.0–9.0)	8.6 (8.0–9.0)	8.3 (8.0–9.0)	7.7 (7.0–8.0)
*Z*	0.652	0.103	0.762	2.635	3.426	2.833	0.762	1.565	2.135	2.588
*P*	0.514	0.918	0.446	**0.008**	**0.001**	**0.005**	0.446	0.118	**0.033**	**0.010**
	**U1**	**U2**	**U3**	**U4**	**U5**	**U6**	**U7**	**U8**	**U9**	**U10**
Ulnar nerve CSA/mm^2^ Mean (P25–P75)	3.7 (3.0–5.0)	4.6 (4.0–5.0)	5.3 (5.0–6.0)	6.3 (5.0–7.0)	6.2 (5.0–7.0)	6.6 (6.0–8.0)	5.3 (5.0–6.0)	6.0 (5.0–6.0)	5.7 (5.0–6.0)	7.1 (5.0–6.0)
Healthy control CSA (mean ± SD)	2.8 (2.0–3.0)	3.9 (3.0–4.0)	4.7 (4.0–6.0)	4.3 (4.0–5.0)	4.8 (4.0–6.0)	5.7 (5.0–7.0)	5.0 (4.0–6.0)	4.3 (4.0–5.0)	4.4 (4.0–5.0)	4.4 (4.0–5.0)
*Z*	3.549	2.078	2.121	4.465	2.977	2.088	0.589	4.415	3.129	4.244
*P*	**0.000**	**0.038**	**0.034**	**0.000**	**0.003**	**0.037**	0.556	**0.000**	**0.002**	**0.000**

*CSA, cross-sectional area; MMN, multifocal motor neuropathy; HC, healthy control. For the median nerve: M1-the outlet of the carpel cannel, M2-the middle point of the wrist crease, M3-the inlet of the carpel cannel, M4-4 cm proximal to the wrist crease, M5-the middle point between the wrist crease and elbow, M6-the entrance into the pronator teres, M7-the elbow, M8-4 cm above the elbow, M9-8 cm above the elbow, M10-the axilla. For the ulnar nerve: U1-the wrist, U2-4 cm above the wrist, U3-departing point from the ulnar artery, U4-alongside the muscle belly of the flexor carpi ulnaris, U5-the outlet of the cubital tunnel, U6-inside the cubital tunnel, U7-the inlet of the cubital tunnel, U8-4 cm proximal to the inlet of the cubital tunnel, U9-8 cm proximal to the inlet of the cubital tunnel, U10-the axilla. The bold values represented statistically significant difference in the cross-sectional area (CSA) of nerve between MMN and HC (*P* < 0.05)*.

### Correlation of maxCSA and the Medical Research Council Sum Score (MRC)

**Figure 2:**
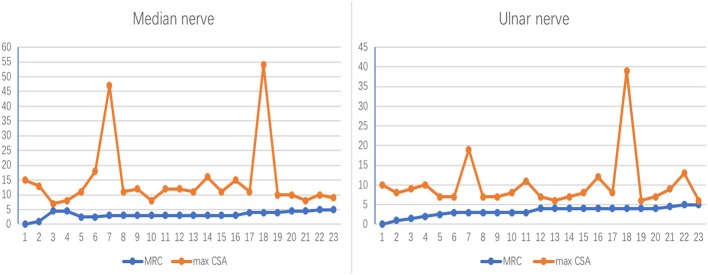
The correlation between nerve maxCSA and MRC. The trend between the maximum nerve cross-sectional area (CSA) of a nerve and the corresponding muscle strength. The abscissa indicates 23 bilateral nerves in 12 patients, one of whom was unable to record the lateral nerve due to amputation; the ordinate represents the nerve cross-sectional area (mm^2^) or muscle strength rating. MRC, Medical Research Council Sum Score; maxCSA, maximum cross-sectional area of a certain nerve.

In the 12 MMN patients, in total 23 median nerves and 23 ulnar nerves had been included because one of MMN patients amputated for work injury. The trend between the maximum nerve CSA of a nerve and the corresponding muscle strength is divided into the following two types (Figure 2): (1) CSA increased and MRC decreased. (2) CSA increased and MRC showed no obvious change.

### Correlation of CSA and CB

With motor NCSs, 12 definite CB and 12 probable CB areas were detected across standard segments of median and ulnar nerves. With ultrasound studies, increased CSA was detected at 36 sites, removing segments that were susceptible to pressed. In the median nerve, the median (P_25_, P_75_) of the maximum CSA of a standard segment was 10.3 (8–12) mm^2^ for those without CB and 21.22 (8.5,38) mm^2^ for those with CB (*Z* = 1.409, *P* = 0.159). In the ulnar nerve, the median (P_25_, P_75_) of the maximum CSA of a standard nerve segment was 7.7 (5.8,7) mm^2^ for those without CB and 6.25 (5,8.25) mm^2^ for those with CB (*Z* = 0.744, *P* = 0.457).

**Figure 3:**
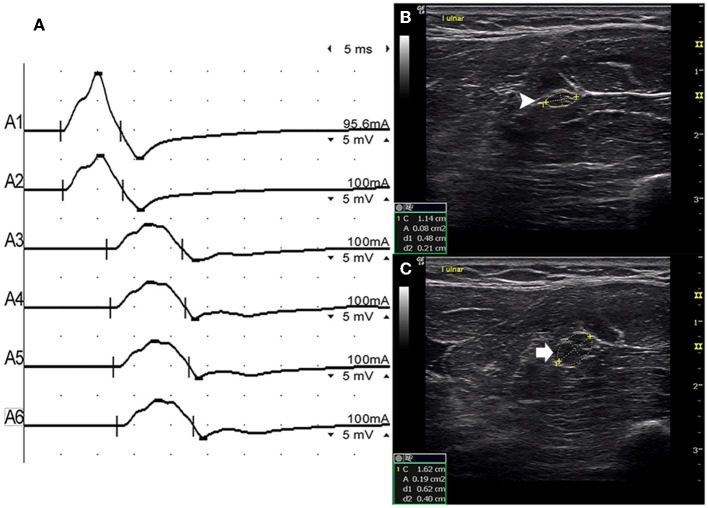
Mode 1. A 28-year-old man with 4 years of progressive asymmetric weakness of the bilateral hands. Examination revealed ulnar nerve innervated muscles of the hand (MRC grade right 3, left 4). An inching technique used across the left forearm segment of the ulnar nerve showed conduction block and CSA enlargement at the same site in one patient with MMN. **(A)** Conduction block was detected between A1 (latency 4.9 ms, duration 8.2 ms, amplitude 14.8 mv, area 38.2 mvms, conduction velocity 66.6 m/s) and A2 (latency 5.2 ms, duration 8.2 ms, amplitude 9.5 mv, area 26.1 mvms, conduction velocity 11.6 m/s). **(B)** The white arrowhead showed that the CSA of A1 was 8 mm^2^. **(C)** The arrow shows that the CSA of A2 was 19 mm^2^ (A1, elbow-6 cm; A2, elbow-4 cm; A3, elbow-2 cm) (Video 1). CSA, cross-sectional area; CB, conduction block; l, left; r, right.

**Figure 4:**
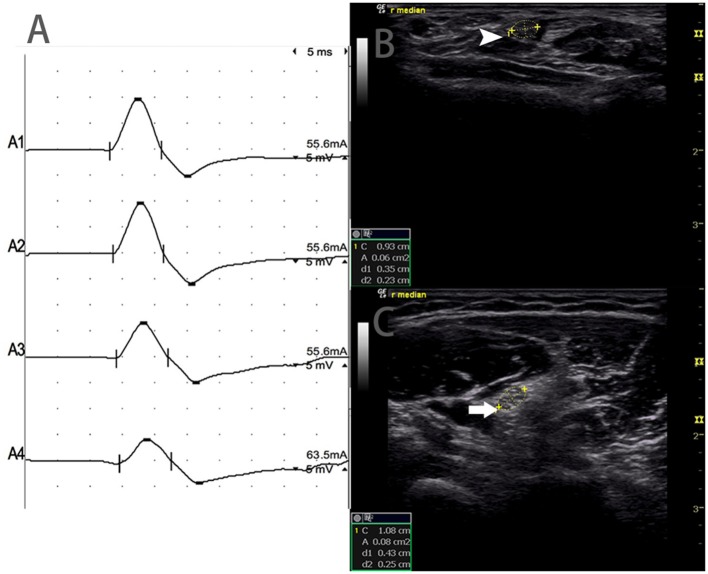
Mode 2. A 46-year-old woman with 11 years of progressive asymmetric weakness of the bilateral upper limbs, with right upper limb MRC grade 3 and left 4, showed conduction block and normal CSA at the same site. **(A)** Conduction blocks were detected between A1 and A2. **(B)** The white arrowhead showed 6 mm^2^ at A1 (Latency 2.8 ms, duration 3.8 ms, amplitude 12.4 mv, area 13.3 mvms, conduction velocity 50.9 m/s) and **(C)** The arrow showed 8 mm^2^ at A2 (Latency 6.8 ms, duration 3.9 ms, amplitude 8.2 mv, area 7.7 mvms, conduction velocity 63 m/s) (A1, wrist; A2, elbow) (Video 2). CSA, cross-sectional area; CB, conduction block; l, left; r, right.

**Figure 5:**
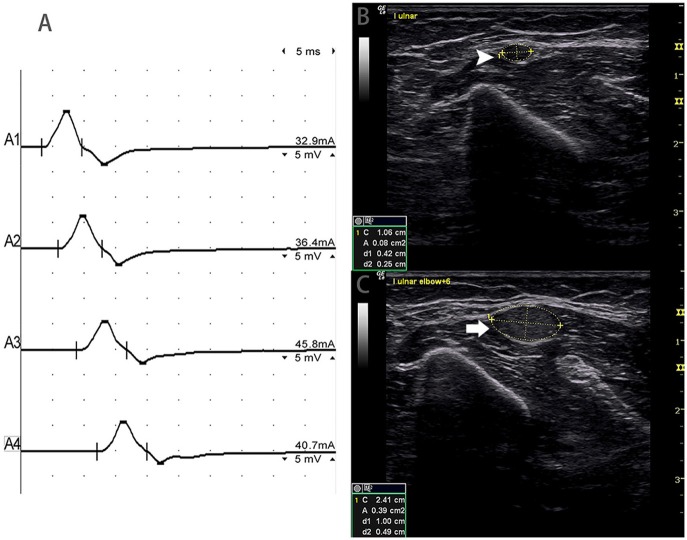
Mode 3. A 51-year-old woman with 4 years of left upper limb weakness and MRC grade 4 showed CSA enlargement without corresponding conduction block. **(A)** Standard segment motor nerve conduction study of the left ulnar nerve. No CB was detected. Nerve ultrasound study across the left upper arm of the ulnar nerve show: **(B)** CSA (white arrowhead) that was 8 mm^2^ at the site of A3 (latency 8.7 ms, duration 8.0 ms, amplitude 8.2 mv, area 19.2 mvms, conduction velocity 48.6 m/s) and **(C)** CSA (arrow) of the site 6 cm proximal to the elbow of the left ulnar that was 39 mm^2^ (latency 12.0 ms, duration 7.9 ms, amplitude 8.4 mv, area 19.5 mvms, conduction velocity 18.2 m/s), but no CB was detected across the same segment (A1, wrist; A2, below elbow; A3, above elbow; A4, axilla) (Video 3). CSA, cross-sectional area; CB, conduction block; l, left; r, right.

There were 9 standard segments with CB and increased CSA (Figure 3, Video 1), 15 segments with CB but normal CSA (Figure 4, Video 2), and 27 segments with increased CSA but no CB (Figure 5, Video 3). The inching technique and consecutive scanning with ultrasound were performed across five segments of which conduction blocks were hardly confirmed by standard segments detection with a partial conduction block. By combining inching techniques and ultrasound, another two more segments showed CBs and increased CSA at the same sites, and 3 segments showed CBs but normal CSA at the same sites.

## Discussion

Electrophysiological studies reflect the physiological features of a nerve, and ultrasound studies reveal the morphological features of a nerve. MMN is one of the ideal models for exploring the correlation between motor CB and CSA, for which only the motor nerve is involved, and CB is the main electrophysiological feature. Although previous studies have reported a correlation between CB and CSA (5, 6, 9), limited sites were observed without nerve continuous scanning under ultrasound, and the lesions in MMN were distributed stochastically and not always at predetermined sites.

In this study, we performed consecutive scanning along the whole nerve to record the CSA at abnormal sites. Additionally, CSAs were measured at 10 predetermined sites. The inching technique was performed if necessary. Although the disease duration in this group of patients with MMN were long and varied, which may have affected the ultrasound and electrophysiological characteristics, we still found three modes of relationships between CSA and CB: CB with corresponding nerve CSA enlargement (Mode 1), CB without corresponding nerve CSA enlargement (Mode 2), and nerve CSA enlargement without corresponding CB (Mode 3). Consequently, CB is not always correlated with increased CSA.

The potential mechanism of these different patterns of correlation between CB and CSA is still unclear. Moreover, the true corresponding pathological manifestations behind nerve enlargement have not been clearly revealed. Hypoechoic enlargement of the nerve may reflect active inflammation and onion bulbs, while nerve enlargement with additional hyperechogenic fascicles/perifascicular tissue may reflect axonal degeneration (10). That is, both axonal and myelin sheath lesions could lead to nerve CSA enlargement (11). With respect to Mode 2, CB without corresponding CSA enlargement can be easily understood. At present, MMN is considered an immunomediated motor neuropathy, which is related to anti-GM1 antibody damage to voltage-gated Na^+^-channels at the node of Ranvier (12, 13). Theoretically, anti-GM1 antibodies trigger direct and complement-dependent damage to axons, leading to conduction block, while there may be no obvious myelin changes. Taylor et al. hypothesized that the antibody attack could be directed at the components of paranodal myelin and found that MMN axonal pathological alteration predominated over myelin pathology (14). In addition, our findings related to normal CSA and CB in MMN might be a consequence of the fact that only single fascicles are enlarged, whereas the main nerve CSA remains unaffected (15).

With respect to Mode 1 (CB with increased CSA), increased CSA in MMN has been reported in magnetic resonance imaging (MRI) (16) and other ultrasound (3) studies. Our finding that patients with MMN had multifocal nerve CSA enlargement and conduction block at the same site along the nerve may imply that at the site of CB, there were not only damaged voltage-gated Na^+^-channels but also some lesions, such as demyelination, edema, and onion bulb formation (6, 17). This mode indicated that CB might be caused by different mechanisms, and MMN may be a syndrome. Not all cases of MMN are caused by anti-GM1 IgM antibodies, and other immunization processes might also be involved resulting in demyelination/remyelination and axonal degeneration/regeneration processes.

The mechanism for Mode 3 needs further exploration. Nerve CSA enlargement without CB in MMN, or even limbs without neurophysiological dysfunction, was also found in other reports (6, 9, 18). We hypothesized that when inflammatory infiltrates, edema, and channel dysfunction occur at the nodes of Ranvier at the early stage, the depolarization threshold of ion channels might remain in a normal range, such that the dysfunction of saltatory stimulus transmission has not yet been reached, and no CB can be detected. In patients with MMN, if increased CSA is detected without CB, the morphological changes of the nerve should also have clinical significance. Consecutive scanning along the nerve and measurements at a greater number of sites to detect morphological changes could increase diagnostic sensitivity for MMN.

In conclusion, three patterns of correlations between CB and CSA existed, and the electrophysiological and morphological changes were not always consistent in MMN. Ultrasound studies could detect more lesions along the nerve in MMN, even without CB. The combination of motor NCS and ultrasound studies could provide more information for clinical diagnosis of MMN.

## Limitations

This was a single parameter comparison, cross-sectional study. The different disease durations of patients with MMN in this study, in addition to their varying heights, weights, could affect nerve CSA and nerve conduction velocity detection. In addition, we only observed whether motor nerve CB presented and whether there were related changes in CSAs on an ultrasound; the potential mechanisms of the different patterns of correlation between CB and CSA require further study. Only nerve CSA, the most important parameter, had been included in this study, so in order to perform more precise research, more index like echo intensity, should be involved in.

## Data Availability Statement

Any data not published within the article are available and will be shared upon request from any qualified investigator.

## Ethics Statement

The ethics committee of Peking Union Medical College Hospital approved our study protocol, and all patients signed an informed consent form in accordance with the Declaration of Helsinki.

## Author Contributions

YL: electrophysiological and ultrasonographic studies, acquisition of data, statistical analysis, and manuscript writing. JN: electrophysiological and ultrasonographic studies and statistical analysis. LC: study concept and design and manuscript editing. TL: data collection and manuscript editing. QD: electrophysiological and ultrasonographic studies. SW and YG: electrophysiological studies. ML: study concept and design, data review, manuscript editing, and critical revision.

### Conflict of Interest

ML received support from Beijing Capital Special Fund (Z171100001017220). The remaining authors declare that the research was conducted in the absence of any commercial or financial relationships that could be construed as a potential conflict of interest.

## Supplementary Material

Video 1 Mode 1: Conduction block and CSA enlargement at the same site in MMN.

Video 2 Mode 2: Conduction block and normal CSA at the same site in MMN.

Video 3 Mode 3: CSA enlargement without corresponding conduction block.

## Abbreviations

MMN: multifocal motor neuropathy
CB: motor nerve conduction block
CSA: cross-sectional area
NCS: motor nerve conduction studies.
